# Study on Molten Pool Flow and Porosity Defects in Laser–Tungsten Inert Gas (TIG) Welding of 4J36 Invar Steel

**DOI:** 10.3390/ma18081824

**Published:** 2025-04-16

**Authors:** Sen Wu, Fei Zhao, Pengfei Wang, Shuili Gong, Zhisheng Wu

**Affiliations:** 1 College of Material Science and Engineering, Taiyuan University of Science and Technology, Taiyuan 030024, China; s202314210420@stu.tyust.edu.cn (S.W.); s202314210410@stu.tyust.edu.cn (P.W.); zswu1963@tyust.edu.cn (Z.W.); 2 Shanxi Innovation Center of Metal Joining and Additive Manufacturing Technology, Taiyuan 030024, China; 3 Aviation Manufacturing Technology Research Institute of China, Beijing 100024, China; gongsl2023@126.com

**Keywords:** 4J36 Invar steel, laser–TIG hybrid welding, flow field, high-speed photography, porosity defect

## Abstract

The Invar steel molten pool is characterized by low fluidity of the molten pool due to high tension, which hinders the escape of gases and exacerbates the formation of porosity defects. In this study, the influences of different welding process parameters, material properties, and U-groove on the flow behavior of the molten pool of laser–tungsten inert gas (TIG) hybrid welding of Invar steel are investigated by numerical simulation and high-speed photography. This research proposes effective measures to suppress porosity defects, such as optimizing process parameters and extending the existence time of the molten pool. In conclusion, this study systematically investigates the dynamic mechanism of the formation of welding defects in 4J36 Invar steel and provides important theoretical support for the optimization of the welding process of 4J36 Invar steel. The results indicate that controlling the laser power at 4–6 kW, welding speed at 0.5–1.0 m/min, and welding current at 150–170 A can stabilize the molten pool flow and keyhole and promote the molten pool flow and gas escape.

## 1. Introduction

4J36 Invar steel is currently mainly used in China’s aerospace, precision instrument, and other fields. Owing to its exceptionally low coefficient of thermal expansion, 4J36 Invar steel is predominantly employed in the manufacturing of molds for composite materials [1,2,3,4,5]. 4J36 Invar steel is an iron–nickel alloy with 36% nickel content, which has the characteristics of low fluidity due to high tension in the liquid metal state. During the welding process, 4J36 Invar steel exhibits a high susceptibility to welding defects, including hot cracks, porosity, and undercut. These defects seriously affect the surface quality of the mold, resulting in low dimensional accuracy of the parts after welding, and there are residual stresses and other phenomena [6,7,8,9]. Currently, domestic and international scholars primarily focus on the following aspects of Invar steel research [10,11,12]: welding process optimization, weld microstructure matching, and post-welding deformation control. However, studies on molten pool fluid dynamics and the mechanisms of welding defect formation remain insufficient. Consequently, the study of the flow state of the molten pool of 4J36 Invar steel can provide a theoretical basis and data support for the mold manufacturing of 4J36 Invar steel [13,14,15].

Laser–tungsten inert gas (TIG) hybrid welding is an efficient and high-quality welding method. It utilizes the characteristics of laser and arc to overcome the shortcomings of both laser welding and arc welding [16,17,18]. Compared with single-heat-source welding, laser–TIG hybrid welding can achieve deeper penetration and larger fusion width. Due to the high tension and low fluidity of 4J36 Invar steel in the liquid metal state, laser–TIG hybrid welding is more suitable for the welding of 4J36 Invar steel.

With the advancement of numerical simulation technology, computational fluid dynamics (CFD) has been successfully applied to welding process research [19]. By visualizing molten pool flow, keyhole dynamics, and defect formation mechanisms, CFD provides a crucial technical approach for analyzing microscopic transient phenomena. Furthermore, the application of compressed sensing technology [20] can effectively reduce noise interference in dynamic scenarios while improving resolution, thereby providing technical support for real-time analysis in high-speed photography with high-frequency sampling. In recent years, domestic and foreign scholars have studied the dynamic behavior of keyholes and the flow behavior of molten pools through numerical simulation and high-speed photography. Cheng et al. [21] investigated the influence of the defocus amount on porosity defects in laser–MIG hybrid welding by simulating the molten pool flow. The formation of a liquid bridge behind the keyhole, under the action of surface tension and molten pool flow, leads to the formation of holes and eventually porosity. Liu et al. [22] studied keyhole behavior and molten pool flow by simulating the laser welding molten pool, focusing on the influence of plate thickness on porosity formation. They found that the keyhole reached a quasi-steady state faster with increasing plate thickness. In addition, the number of bubbles is significantly reduced at low laser power and high welding speeds. Muhammad et al. [23] focused on the effect of laser power and welding speed on the evolution of the keyholes and molten pool flow. It was found that eddies will form inside the molten pool when the laser power is greater than 6 KW. In addition, penetration decreases as the welding speed increases. Li et al. [24] used high-speed photography to capture the metal transfer behavior of a 30 mm thick Q235 steel strip with a narrow gap for Y-shaped bevels. The smaller the groove angle, the lower the frequency of metal transfer. Park et al. [25] recorded the arc morphology and molten pool flow of V-bevels under different welding parameters with the help of high-speed photography technology. The methods of numerical simulation and high-speed photography technology provide an in-depth understanding of the molten pool flow behavior during the welding process and allow real-time monitoring and optimization of the welding process. The aforementioned studies mainly concentrate on the welding process of conventional steels, and there are fewer reports related to Invar steel. The formation mechanism of porosity defects remains unclear, especially under the low-fluidity condition characteristic of the molten pool.

This study investigates the dynamic influence mechanism of molten pool flow and porosity defects in laser–TIG hybrid welding of Invar steel by simulating molten pool behavior and capturing flow characteristics in U-groove using high-speed photography. This study provides critical theoretical support for optimizing Invar steel welding processes by systematically investigating the effects of welding parameters, material properties, and U-groove geometry on the flow behavior of the molten pool.

## 2. Materials and Methods

### 2.1. Materials

**Figure 1:**
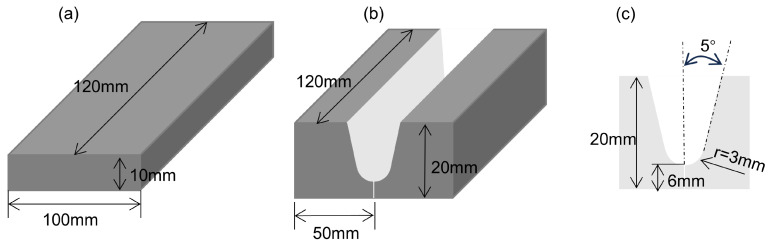
4J36 base metal groove schematic diagram: (**a**) 10 mm, no groove; (**b**) 20 mm, U-groove; (**c**) schematic diagram of U-groove dimensions.

The material used in this study is a 4J36 Invar steel plate with a thickness of 10 mm and 20 mm. Its specific dimensions are shown in Figure 1. Invar 36 is selected as the filler metal with a 1.2 mm diameter. Its chemical composition is the same as that of the base metal. The chemical composition of the base material is provided in Table 1. High-purity argon (99.999%) with a flow rate of 20 L/min was used during welding. High-purity argon gas (99.999%) was used as the shielding gas at a flow rate of 20 L/min during welding. Prior to welding, the base metal was wiped with acetone to remove oil, grease, and other contaminants. It was then ground with 180-grit silicon carbide sandpaper to eliminate surface oxide layers and impurities. Subsequently, the base metal was wiped again with acetone to ensure complete removal of any residual oil or grease. Finally, the surface was maintained in a dry and clean condition, and welding was performed immediately.

### 2.2. Methods

**Figure 2:**
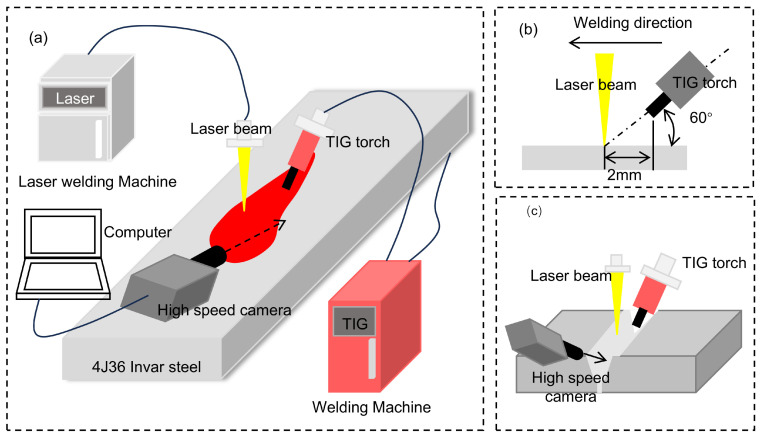
Laser–TIG hybrid welding system: (**a**) experimental equipment; (**b**) welding position; (**c**) high-speed camera position.

As shown in Figure 2a, the experimental system is composed of the laser (TruDisk 8000, TRUMPF, Ditzingen, Germany), the welding machine (TransTig 2200, FRONIUS, Austria), and the high-speed camera (Phantom v1840, Vision Research, Wayne, USA). The maximum output power of the laser was 8000 W, the wavelength was 1030 nm, the diameter of the fiber was 200 µm, and the spot diameter was 0.3 mm. The welding machine was selected in DC mode, the height of the tungsten electrode was 2 mm, the distance between the laser and the tungsten electrode was 2 mm, and the angle between the welding torch and the plane of the workpiece was 60°. The high-speed camera captured the process with a frame rate of 2000 fps, an exposure time of 1 µs, and a pixel resolution of 2560 × 1440.

The base metal of 10 mm thick Invar steel was used for plate cladding experiments. Welding parameters are shown in Table 2. The experimental results were compared with the simulation results. The welded joints were observed under a super depth of field microscope after sectioning, grinding, polishing, and etching to examine its microstructure.

The base metal of 20 mm thick Invar steel was used to investigate the effect of the U-groove on the flow of the molten pool. The specific welding parameters are shown in Table 2. The high-speed camera was positioned directly in front of the welding direction at about 45 degrees to ensure that a clear image of the molten pool was captured. During the welding process, an infrared laser light was employed as the backlight source, with the focus position adjusted to the tungsten electrode tip. Prior to welding, the distance between the high-speed camera and the tungsten electrode was adjusted, followed by the adjustments of the exposure time and the lens aperture to focus on the tungsten electrode tip until an appropriately sized image of the tungsten electrode appeared in the center of the screen. Additionally, a filter was installed in front of the high-speed camera lens to attenuate the arc light until a clear image was visible on the screen.

### 2.3. Molten Pool Numerical Simulation Analysis

#### 2.3.1. Basic Assumptions

For the laser–TIG hybrid welding of 4J36 Invar steel, the following assumptions were made.

(1) The liquid metal in the computational domain is an incompressible Newtonian fluid in laminar flow.
(2) The influence of shielding gas and metal transfer is neglected during the welding process.
(3) The driving forces in the molten pool mainly include the recoil pressure, surface tension, buoyancy, gravity, arc pressure, and electromagnetic force.

#### 2.3.2. Control Equations

In laser–TIG welding processes, heat and mass transfer, as well as fluid flow, adhere to the three conservation equations of mass, energy, and momentum [26,27,28].

(1) The mass conservation equation is

(1) $$\frac{\partial \rho }{\partial t}+\frac{\partial (\rho u)}{\partial x}+\frac{\partial (\rho v)}{\partial y}+\frac{\partial (\rho w)}{\partial z}=0$$

where ρ is the material density, t is the time, and u,v and w are the flow velocity of liquid metal in x,y and z directions, respectively.

(2) The conservation of momentum equation is

(2) $$\frac{\partial (\rho u)}{\partial t}+\frac{\partial (\rho uu)}{\partial x}+\frac{\partial (\rho uv)}{\partial y}+\frac{\partial (\rho uw)}{\partial z}=-\frac{\partial P}{\partial x}+\frac{\partial }{\partial x}\left(\mu \frac{\partial u}{\partial x}\right)+\frac{\partial }{\partial y}\left(\mu \frac{\partial u}{\partial y}\right)+\frac{\partial }{\partial z}\left(\mu \frac{\partial u}{\partial z}\right)+S_{u}$$

(3) $$\frac{\partial (\rho v)}{\partial t}+\frac{\partial (\rho vu)}{\partial x}+\frac{\partial (\rho vv)}{\partial y}+\frac{\partial (\rho vw)}{\partial z}=-\frac{\partial P}{\partial y}+\frac{\partial }{\partial x}\left(\mu \frac{\partial v}{\partial x}\right)+\frac{\partial }{\partial y}\left(\mu \frac{\partial v}{\partial y}\right)+\frac{\partial }{\partial z}\left(\mu \frac{\partial v}{\partial z}\right)+S_{v}$$

(4) $$\frac{\partial (\rho w)}{\partial t}+\frac{\partial (\rho wu)}{\partial x}+\frac{\partial (\rho wv)}{\partial y}+\frac{\partial (\rho ww)}{\partial z}=-\frac{\partial P}{\partial z}+\frac{\partial }{\partial x}\left(\mu \frac{\partial w}{\partial x}\right)+\frac{\partial }{\partial y}\left(\mu \frac{\partial w}{\partial y}\right)+\frac{\partial }{\partial z}\left(\mu \frac{\partial w}{\partial z}\right)+S_{w}$$

where P is the pressure within the fluid, μ is the dynamic viscosity coefficient of the liquid metal, and $S_{u},{\;S}_{v}$ and $S_{w}$ are the source terms in the momentum equation, respectively.

(5) $$S_{u}={{-S}_{x}+P}_{rx}+F_{ex}$$

(6) $$S_{v}={-S}_{y}+P_{ry}+F_{ey}$$

(7) $$S_{w}=-S_{z}+P_{rz}+F_{ez}+F_{b}+P_{A}$$

Here, $S_{x},{\;S}_{y}$ and $S_{z}$ are the components of momentum change on x,y and z, respectively; $P_{rx},P_{ry}$ and $P_{rz}$ are the components of recoil pressure on x,y and z, respectively; $F_{ex},F_{ey}$ and $F_{ez}$ are the components of electromagnetic force on x,y and z, respectively; $F_{b}$ is the buoyancy; and $P_{A}$ is the arc pressure.

The enthalpy–porosity [29] method is used to simulate the solidification–melting problem, and the momentum change in the solid–liquid paste region can be calculated as follows:

(8) $$S=A_{mush}\frac{{\left(1-f_{1}\right)}^{2}}{\left({f_{1}}^{3}+\varepsilon \right)}\vec{v}$$

where $A_{mush}$ is the paste zone parameter, $f_{1}$ is the liquid phase volume fraction, ε is the very small value that prevents the denominator from being 0, and $\vec{v}$ is the fluid velocity vector.

During the welding process, the flow of the molten pool is mainly influenced by recoil pressure, electromagnetic force, arc pressure, buoyancy, and surface tension [30,31,32,33,34].

Recoil pressure can be calculated as

(9) $$P_{r}=0.54P_{0}exp\left(∆H_{v}\frac{T-T_{b}}{RTT_{b}}\right)$$

where $P_{0}$ is the environmental pressure, $∆H_{v}$ is the latent heat of evaporation of the material, T is the temperature of the keyhole wall, $T_{b}$ is the boiling point of the molten metal, and R is the ideal gas constant.

For electromagnetic force, $F_{ex},{\;F}_{ey}$ and $F_{ez}$ are the components of electromagnetic force on x, y and z, respectively:

(10) $$F_{ex}=-\frac{\mu_{0}I^{2}}{4\pi^{2}\sigma_{j}^{2}r}exp\left(-\frac{r^{2}}{2\sigma_{j}^{2}}\right)\left[1-exp\left(-\frac{r^{2}}{2\sigma_{j}^{2}}\right)\right]{\left(1-\frac{z}{L}\right)}^{2}\frac{x}{r}$$

(11) $$F_{ey}=-\frac{\mu_{0}I^{2}}{4\pi^{2}\sigma_{j}^{2}r}exp\left(-\frac{r^{2}}{2\sigma_{j}^{2}}\right)\left[1-exp\left(-\frac{r^{2}}{2\sigma_{j}^{2}}\right)\right]{\left(1-\frac{z}{L}\right)}^{2}\frac{y}{r}$$

(12) $$F_{ez}=\frac{\mu_{0}I^{2}}{4\pi^{2}Lr^{2}}{\left[1-exp\left(-\frac{r^{2}}{2\sigma_{j}^{2}}\right)\right]}^{2}\left(1-\frac{z}{L}\right)$$

where $\mu_{0}$ is the magnetic permeability, L is the thickness of the workpiece, I is the current magnitude, $\sigma_{j}$ is the current density distribution coefficient, and $v_{0}$ is the welding speed. In addition, $r=\sqrt{{\left(x-v_{0}t\right)}^{2}+y^{2}}$.

Arc pressure can be calculated as follows:

(13) $$P_{A}\left(x,y\right)=C\frac{3\mu_{0}I^{2}}{2\pi^{2}\left(a_{j1}+a_{j2}\right)b_{j}}exp\left(-\frac{3{\left(x-v_{0}t\right)}^{2}}{a_{j1}^{2}}-\frac{3y^{2}}{b_{j}^{2}}\right)x\geq 0$$

(14) $$P_{A}\left(x,y\right)=C\frac{3\mu_{0}I^{2}}{2\pi^{2}\left(a_{j1}+a_{j2}\right)b_{j}}exp\left(-\frac{3{\left(x-v_{0}t\right)}^{2}}{a_{j2}^{2}}-\frac{3y^{2}}{b_{j}^{2}}\right)x<0$$

where $\mu_{0}$ is the permeability, C is the calculation coefficient, $a_{j1},a_{j2},b_{j}$ are the arc pressure distribution parameters, and $v_{0}$ is the welding speed.

Buoyancy can be calculated as

(15) $$F_{b}=\rho g\beta \left(T-T_{0}\right)$$

where ρ is the liquid metal density, β is the coefficient of thermal expansion, g is the gravitational acceleration, $T_{0}$ is the material melting point, and T is the liquid metal temperature.

Surface tension can be calculated as

(16) $$F_{s}=\gamma k$$

where γ is the surface tension and k is the radius of curvature of the free surface of the molten pool.

(1) The conservation of energy equation is

(17) $$\frac{\partial (\rho H)}{\partial t}+\frac{\partial (\rho uH)}{\partial x}+\frac{\partial (\rho vH)}{\partial y}+\frac{\partial (\rho wH)}{\partial z}=\frac{\partial }{\partial x}\left(K\frac{\partial T}{\partial x}\right)+\frac{\partial }{\partial y}\left(K\frac{\partial T}{\partial y}\right)+\frac{\partial }{\partial z}\left(K\frac{\partial T}{\partial z}\right)+S_{E}$$

where K is the thermal conductivity of the material, T is the temperature, H is the enthalpy of mixing, and $S_{E}$ is the source term in the energy equation.

(18) $$S_{E}=q_{l}+q_{t}$$

Here, $q_{l}$ is the energy input of the laser heat source and $q_{t}$ is the energy input of the arc heat source.

Heat source modeling:

Models of Gaussian heat sources for lasers and double ellipsoids for arcs are described as follows [35,36,37].

For laser heat source modeling,

(19) $$q(x,y,z)=\frac{3c_{s}Q}{\pi H\left(1-\frac{1}{e^{3}}\right)}exp\left[\frac{-3c_{s}}{log\left(\frac{H}{z}\right)}(x^{2}+y^{2})\right]$$

(20) $$c_{s}=\frac{3}{R_{0}^{2}}$$

where *H* is the height of the heat source; *Q* is the heat input rate; and $R_{0}$ is the radius of the heat source.

For TIG heat source modeling,

(21) $$q_{f}(x,y,z)=\frac{12\sqrt{3}f_{f}Q}{a_{f}b_{h}c_{h}\pi \sqrt{\pi }}exp\left(-\frac{3x^{2}}{a_{f}^{2}}-\frac{3y^{2}}{b_{h}^{2}}-\frac{3z^{2}}{a_{h}^{2}}\right)x\geq 0$$

(22) $$q_{r}(x,y,z)=\frac{12\sqrt{3}f_{r}Q}{a_{r}b_{h}c_{h}\pi \sqrt{\pi }}exp\left(-\frac{3x^{2}}{a_{r}^{2}}-\frac{3y^{2}}{b_{h}^{2}}-\frac{3z^{2}}{a_{h}^{2}}\right)x<0$$

where $q_{f}$ and $q_{r\;}$ are the heat flow distributions in the front- and back-half ellipsoid, respectively; $b_{h}$ and $c_{h}$ are the other two semi-axes of the front- and back-half ellipsoid, respectively; $a_{f}$ and $a_{r}$ are the half-axes of the front- and back-half ellipsoid, respectively; and $f_{r}$ and $f_{r}$ are the share of the heat input in the body of the front- and back-half ellipsoid, respectively. Additionally, $f_{f}+f_{r}=1$.

#### 2.3.3. Boundary Condition

(1) Initial conditions are

(23) $$T=T_{0},u=v=w=0$$

where T is the temperature of the workpiece, $T_{0}$ is the ambient temperature, and u,v and w are the components of the fluid velocity in the direction of x, y and z, respectively.

(2) Boundary conditions

Top surface of workpiece:

(24) $$-K\frac{\partial T}{\partial n}=q_{l}+q_{t}-q_{conv}-q_{rad}$$

(25) $$q_{conv}=h\left(T-T_{0}\right)$$

(26) $$q_{rad}=\sigma \varepsilon \left(T^{4}-T_{0}^{4}\right)$$

Other sides of the workpiece:

(27) $$-K\frac{\partial T}{\partial n}=-q_{conv}-q_{rad},u=v=w=0$$

Symmetry plane:

(28) $$-K\frac{\partial T}{\partial n}=0,\frac{\partial u}{\partial y}=\frac{\partial w}{\partial y}=0,v=0$$

Here, K is the thermal conductivity of the material, $\frac{\partial T}{\partial n}$ is the temperature gradient, $q_{l}$ is the laser heat source, $q_{t}$ is the arc heat source, $q_{conv}$ is the convective heat dissipation, $q_{rad}$ is the radiative heat dissipation, h is the convective heat transfer coefficient, $T_{0}$ is the ambient temperature, σ is Boltzmann’s constant, and ε is the thermal emissivity.

#### 2.3.4. Model Setup

**Figure 3:**
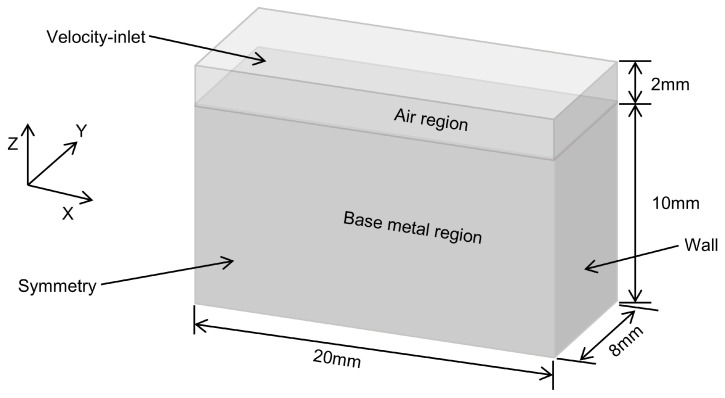
Model.

To simplify the calculations, the model was designed to be half. As shown in Figure 3, the dimensional size of the model is 20 mm × 8 mm × 12 mm (xyz). The upper part of the model is the air region (0 ≤ z ≤ 2) and the lower part is the base material region (−10 ≤ z ≤ 0). The laser beam is directed vertically to the Y-X plane. The welding direction is along the positive direction of the X-axis and perpendicular to the Y-Z plane of the workpiece.

#### 2.3.5. Material Properties

The physical properties of the materials to be used in the simulation process are shown in Table 3.

#### 2.3.6. Model Validation

**Figure 4:**
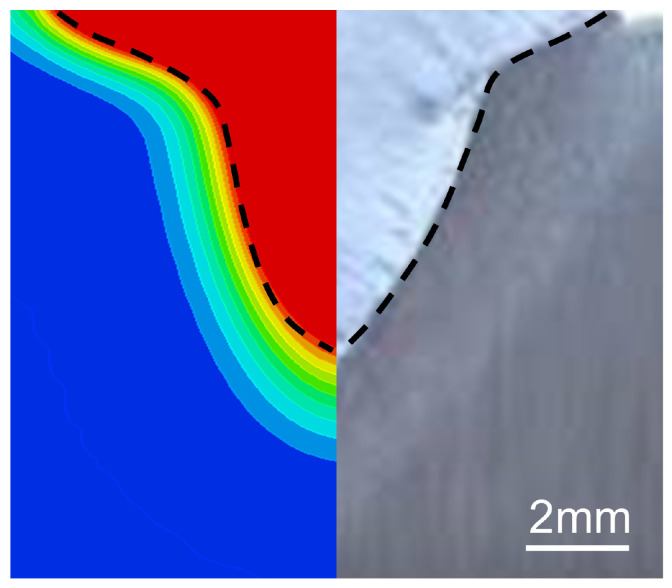
Comparison of numerical simulation and experimental results.

The comparison of numerical simulation results with experimental results is presented in Figure 4. The welding parameters are P = 6000 W, V = 1.2 m/min, and I = 100 A. It can be found that the two molten pool shapes are basically identical. However, there are minor differences in details due to a simplification of the welding process, which does not affect the final simulation results.

## 3. Results

### 3.1. Flow Behavior of Molten Pool

**Figure 5:**
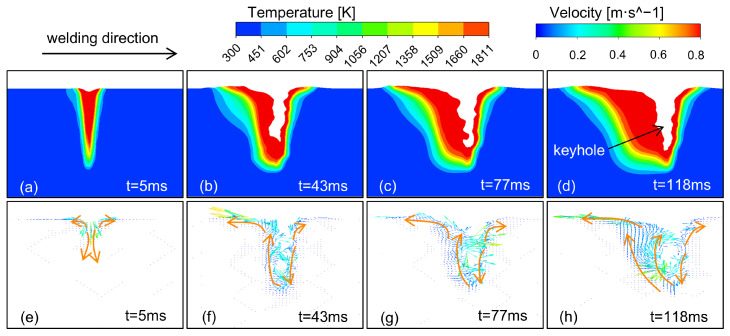
Dynamic distribution of temperature and flow fields in the molten pool: Temperature field: (**a**) t = 5 ms; (**b**) t = 43 ms; (**c**) t = 77 ms; (**d**) t = 118 ms; Flow field: (**e**) t = 5 ms; (**f**) t = 43 ms; (**g**) t = 77 ms; (**h**) t = 118 ms.

Figure 5 shows the temperature field and flow field of the laser–TIG welding pool of the 4J36 Invar steel at different moments. The welding parameters are P = 6000 W, V = 1.2 m/min, and I = 100 A.

Figure 5 shows the formation process of the molten pool. As the base material is continuously heated by the thermal energy source, it melts to form the molten pool, and the liquid metal in the molten pool flows to the surrounding area under the action of arc force and surface tension. Liquid metal is vaporized by the laser to generate recoil pressure, which drives the flow of liquid metal and forms a keyhole. Furthermore, the formation of the keyhole leads to intensified flow of the molten pool and further expansion of its range. The liquid metal around the keyhole flows down along the front wall to the bottom under the action of the recoil pressure and then flows upward along the wall. The liquid metal on the surface of the molten pool flows to the tail under the action of arc force and surface tension.

The laser heat source is mainly applied to the front half, with deep penetration and a keyhole. The TIG heat source acts on the second half, and the molten pool is shallower and longer. The welding process reaches a quasi-steady state when the penetration and width remain unchanged.

### 3.2. Mechanism of Porosity Formation

Due to the high tension and low fluidity of 4J36 Invar steel in a liquid state, porosity defects are prone to occur during the welding. The common types of porosity mainly include two kinds [38,39]: metallurgical porosity and process porosity.

Metallurgical porosity is primarily caused by the decomposition of water in the molten pool during welding, which generates a significant amount of hydrogen. As the molten pool cools, hydrogen has no time to escape and forms hydrogen porosity. Process porosity is related to the welding process parameters. Improper setting of the welding parameter can lead to keyhole instability, causing keyhole collapse and the subsequent formation of bubbles. These bubbles solidify in the molten pool to form a porosity defect. To suppress the formation of process porosity, it is essential to investigate the flow behavior of the molten pool and optimize the welding process parameters. The formation mechanism of porosity defects is analyzed from the dynamic behavior of the molten pool, material properties, and welding process parameters.

#### 3.2.1. Influence of the Molten Pool Dynamics Behavior

**Figure 6:**
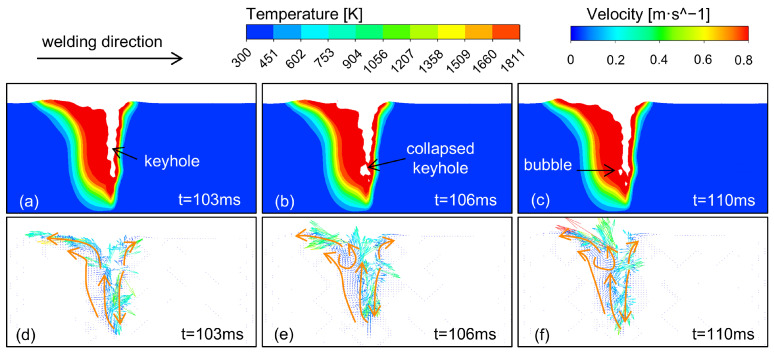
Formation process of porosity defects: Temperature field: (**a**) t = 103 ms; (**b**) t = 106 ms; (**c**) t = 110 ms; Flow field: (**d**) t = 103 ms; (**e**) t = 106 ms; (**f**) t = 110 ms.

Instability of the keyhole and molten pool flow are the direct causes of the formation of porosity defects. Figure 6 is a schematic diagram of the temperature field and flow field during the porosity formation process. The welding parameters are P = 8000 W, V = 1.2 m/min, and I = 100 A.

When the keyhole shape tends to be narrow, the front and back walls of the keyhole adhere to each other under the surface tension, as shown in Figure 6. The flow of liquid metal at the keyhole is violent, resulting in the instability and collapse of the keyhole structure. When the keyhole collapses, the internal gas is enveloped by the surrounding liquid metal, forming a bubble. When the keyhole is reopened, some bubbles escape, whereas the bubbles that do not escape are transported by the flow of the molten pool. Moreover, the bottom of the molten pool and the molten pool wall have the slowest flow rates. Bubble flow to the edge is difficult to avoid, finally forming porosity defects.

The formation of porosity is also related to the rapid solidification behavior of the molten pool. The cooling rate of the molten pool is high, resulting in the difficulty of bubbles escaping at the solidification front. Even if the bubbles reach the surface, the higher surface tension prevents their escape, ultimately forming porosity defects.

#### 3.2.2. The Influence of 4J36 Invar Steel Material Properties

**Figure 7:**
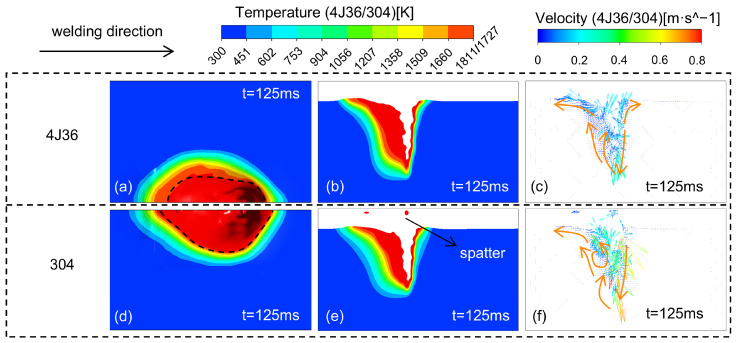
Molten pool with different materials: 4J36: (**a**) The surface of the molten pool; (**b**)Longitudinal section of the molten pool; (**c**) Flow field; 304: (**d**) The surface of the molten pool; (**e**) Longitudinal section of the molten pool; (**f**) Flow field.

The high surface tension of the 4J36 Invar steel molten pool results in low fluidity of the molten pool, which affects the formation of porosity. Figure 7 is a comparative chart of the temperature field and flow field of the molten pool of 4J36 Invar steel and 304 stainless steel. The welding parameters are P = 6000 W, V = 1.2 m/min, and I = 100 A.

As shown in Figure 7, the molten pool of 4J36 Invar steel exhibits a narrower width and an average flow velocity of 0.1 m/s. In comparison, the molten pool of 304 stainless steel is wider, and the average flow velocity is 0.4 m/s. Although the molten pool flow behaviors of the two materials show similarity as a whole, there are significant differences in their molten pool morphology and flow velocity. These results show that the fluidity of the 304 stainless steel molten pool is significantly better than that of the 4J36 Invar steel, further supporting the characterization of the 4J36 Invar steel molten pool with high tension and low fluidity. Moreover, due to the insufficient fluidity of the 4J36 Invar steel molten pool, it is difficult for gases to escape from the molten pool, making it prone to porosity.

Therefore, more strict control of the welding parameters and the purity of the shielding gas are required when welding 4J36 Invar steel to reduce the porosity defects.

#### 3.2.3. Influence of Welding Process Parameters

**Figure 8:**
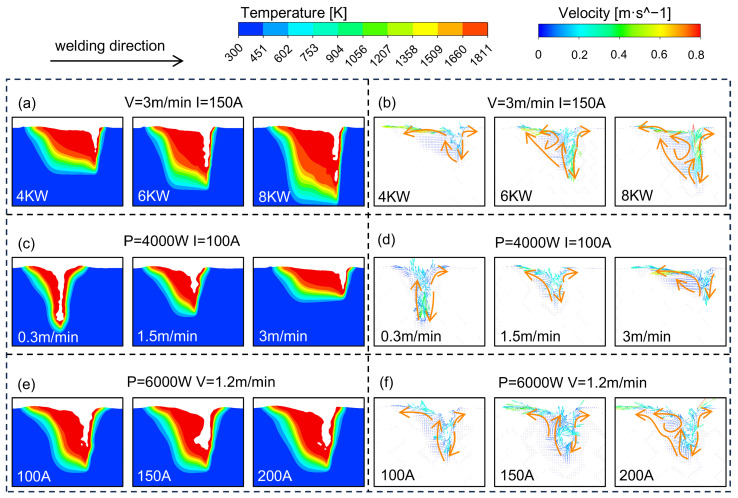
Comparison of molten pool profiles from high-speed photography and numerical simulation: (**a**) The influence of laser power on the temperature field; (**b**) The influence of laser power on the flow field; (**c**) The influence of welding speed on the temperature field; (**d**) The influence of welding speed on the flow field; (**e**) The influence of welding current on the temperature field; (**f**) The influence of welding current on the flow field.

Different process parameters influence the formation of porosity by controlling the flow in the molten pool. Figure 8 shows the temperature field and flow field of the molten pool for different welding parameters. The specific welding parameters are shown in Table 4.

Figure 8a,b show the influence of different laser powers on the flow behavior of the molten pool. With the increase in laser power from 4 KW to 8 KW, the improvement of energy input leads to the growth of penetration and keyholes. When the laser power is increased to 8 kW, the lower and middle parts of the keyhole are more affected by the increase in the recoil pressure, resulting in higher fluctuations in the keyhole wall. The frequency of keyhole collapse also increases with the increase in surface tension gradient. Simultaneously, the enhancement of Marangoni convection leads to an increase in the flow velocity of the molten pool, generating more bubbles.

Figure 8c,d show the influence of different welding speeds on the flow behavior of the molten pool. With the increase in welding speed, the time of heat source acting on the unit area is shortened, which leads to a decrease in energy absorbed by the base metal, resulting in a decrease in both the penetration and the keyhole depth, and the stability of the keyhole is also reduced. The flow velocity in the molten pool is relatively fast under high welding speeds, and the cooling speed of the molten pool is also accelerated, which exacerbates the phenomenon of bubble retention.

Figure 8e,f show the influence of different welding currents on the flow behavior of the molten pool. As the welding current increases, not only does the arc heat input increase, but the arc force is also enhanced, which is beneficial to the expansion and sustainability of the keyhole. When the welding current is increased to 200 A, the impact of arc force on the keyhole wall is intensified, which accelerates the surface flow velocity of the molten pool, thus affecting the stability of the keyhole. Moreover, the variation in welding current will also cause a change in surface tension gradient, which further affects the morphology and stability of the keyhole.

Laser power, welding speed, and welding current determine the stability characteristics of the keyhole and the flow behavior of the molten pool by controlling the key factors such as energy input, heat source action time, molten pool stress state, and dynamic behavior of the keyhole wall. The complex interaction between these factors results in significant differences in the stability of the keyhole and the behavior of the molten pool under different process parameter conditions, which in turn has a significant impact on the quality and performance of the weld.

The combination of high laser power and low welding speed significantly enhances energy input, which not only increases the depth of the molten pool and keyhole but also supports the formation and stable maintenance of the keyhole. The synergistic effect of high laser power and moderate welding current significantly enhances energy input, leading to increased penetration and keyhole depths. Meanwhile, the enhancement of arc force helps to maintain the open state and stability of the keyhole. The process combination of low welding speed and high welding current prolongs the heat source duration and enhances the arc heat input, which is conductive and increases the penetration and keyhole depths.

### 3.3. Microstructure Analysis of Invar Steel Laser–TIG Hybrid Welded Joint

**Figure 9:**
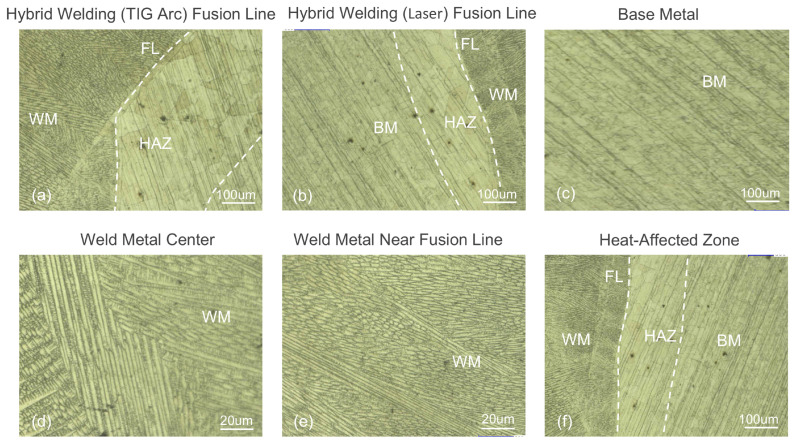
Microstructure of Invar steel laser–TIG hybrid welded joint: (**a**) hybrid welding (TIG arc) fusion line; (**b**) hybrid welding (laser) fusion line; (**c**) base metal; (**d**) weld metal center; (**e**) weld metal near fusion line; (**f**) heat-affected zone.

As shown in Figure 9, the microstructure of the Invar steel laser–TIG hybrid welded joint shows significant differences in regional features. The base metal exhibits a homogeneous microstructure with fine austenite grains. The grains in the heat-affected zone become significantly larger due to thermal cycling. The heat-affected zone in the arc-treated area is wider and shows more severe grain coarsening than that in the laser-treated area, and the grain size decreases in a gradient from the fusion line to the base metal. Near the fusion line, the weld’s columnar grains grow perpendicular to the fusion boundary. The weld center exhibits a mixed microstructure with randomly oriented equiaxed grains and longitudinally grown columnar grains.

### 3.4. Porosity Inhibition Measures

In view of the high tension and low fluidity of 4J36 Invar steel, the existence time of the molten pool can be prolonged by optimizing the welding parameters so as to promote the escape of bubbles.

(1) The base metal must be thoroughly cleaned prior to welding. Surface oil, grease, and other contaminants are first removed with organic solvents such as acetone, alcohol, and others. Then, the surface oxides are removed by sanding with a stainless steel wire brush or sandpaper. Finally, the surface is thoroughly cleaned again of oil, grease, dust, or other organic contaminants with organic solvents such as acetone, alcohol, and others. The base metal surface must be maintained in a completely dry condition prior to welding to prevent moisture retention. The cooling rate of the molten pool can be effectively reduced through preheating treatment of the base metal.
(2) When the laser power is increased from 4 kW to 8 kW, the enhanced heat input significantly increases the penetration depth and enlarges the keyhole size. However, when the laser power exceeds 6 kW, it tends to induce keyhole instability. Therefore, maintaining the power within the 4–6 kW range is recommended. When the welding speed decreases from 3 m/min to 0.3 m/min, the prolonged thermal interaction promotes molten pool flow expansion. However, the welding speed exceeding 1.5 m/min increases porosity defects. Thus, an optimal speed range of 0.5–1.5 m/min is recommended. Increasing the welding current from 100 A to 200 A widens the keyhole and enhances molten pool fluidity. Furthermore, maintaining the current within the 150–200 A range effectively mitigates the risk of keyhole collapse. The coordinated optimization of multiple parameters enables a stabilized welding process.

### 3.5. Flow Behavior of Molten Pool in U-Groove

Based on the numerical simulation of the analysis of the flow behavior of a 10 mm thick Invar steel molten pool, it is found that the material properties and process parameters can cause variations in the flow behavior of the molten pool. In practical welding engineering, variations in plate thickness and groove geometry can significantly alter the dynamic characteristics of the molten pool. To validate the numerical model under complex working conditions and directly capture the influence of U-groove geometry on molten pool flow behavior, this study employs high-speed imaging to observe the weld pool dynamics during U-groove welding of 20 mm thick plates. Analysis of molten pool morphology and liquid metal flow in U-groove weldments can reveal the interaction mechanisms between groove geometry and pool flow behavior.

**Figure 10:**
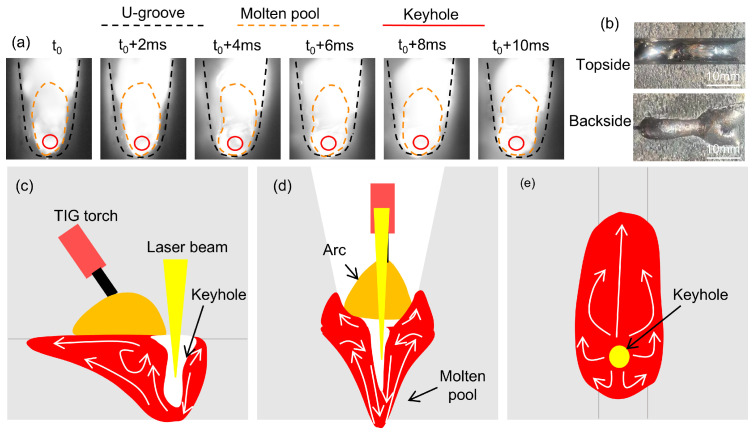
Molten pool under U-groove: (**a**) high-speed photographic images; (**b**) weld bead; (**c**) longitudinal section; (**d**) transverse section; (**e**) top surface.

Figure 10a shows a high-speed photographic image of the molten pool in the backing weld. The welding parameters are P = 6000 W, V = 1.0 m/min, and I = 150 A. Under this parameter, the bottom of the groove can be penetrated, and the molten pool is ellipsoidal overall, as shown in Figure 10a,b.

Figure 10c demonstrates that the molten pool flow behavior is similar in both backing welding and flat-plate surfacing welding. The keyhole forms under recoil pressure, and liquid metal flows along the keyhole to the bottom of the molten pool and then flows along the slope wall to the surrounding area and upward under the arc force and surface tension. Figure 10d shows that the cross-section of the molten pool exhibits a symmetric circular flow pattern. The liquid metal flows down to the bottom along the keyhole and then flows up along the wall. Compared with the molten pool of surfacing, the liquid metal on the surface of the molten pool of the backing weld is limited by the side walls and fills up along the walls.

### 3.6. Influence of Process Parameters on Flow Behavior of Molten Pool in U-Groove

**Figure 11:**
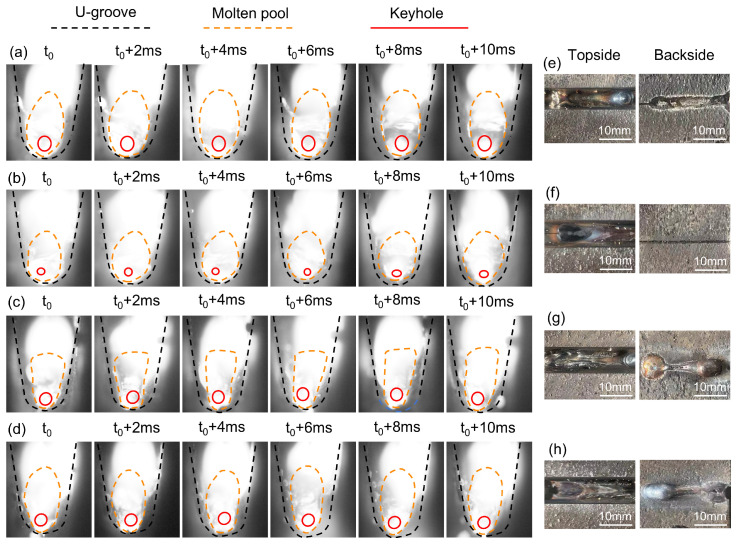
Molten pool images and the weld beads at different welding parameters: (**a**) images of the molten pool of the reference parameters P = 5 KW, V = 1 m/min, and I = 150 A; (**b**) images of the molten pool at lower laser power at P = 4 KW; (**c**) images of the molten pool at faster welding speed at V = 1.2 m/min; (**d**) images of molten pool at higher welding current at I = 170A; (**e**) weld beads of reference parameters at P = 5 KW, V = 1 m/min, and I = 150 A; (**f**) weld beads at lower laser power at P = 4 KW; (**g**)weld beads at faster welding speed at V = 1.2 m/min; (**h**)weld beads at higher welding current at I = 170 A.

Figure 11 shows the molten pool images and their weld forming pictures under different welding parameters.

As shown in Figure 11a,b, the molten pool morphology under a welding speed of 1.0 m/min and a welding current of 150 A is presented for laser powers of 5 kW and 4 kW, respectively. A laser power of 5 kW is sufficient to meet the energy requirements for backing weld. With the increase in laser power, a more stable conductive channel can be provided for the arc, which improves the arc stability. The molten pool area is larger at 5 kW than at 4 kW, but both are oval in shape. Figure 11a,c show the molten pool morphology at a laser power of 5 kW and a welding current of 150 with welding speeds of 1.0 m/min and 1.2 m/min, respectively. With the increase in welding speed, the arc stability decreases and is easily attracted by the bevel wall, and the length of the molten pool is stretched. Simultaneously, higher welding speeds lead to a lack of fusion in the root pass. Figure 10a,d show the molten pool morphology at a laser power of 5 kW and a welding speed of 1.0 m/min with welding currents of 150 A and 170 A, respectively. Compared with 150 A, the welding current of 170 A increases the heat input and melts more metal. It also promotes the flow of the molten pool, allowing the liquid metal to fill the walls on both sides of the bevel. However, welding current exceeding 170 A may result in arc deflection toward the U-shaped wall.

**Figure 12:**
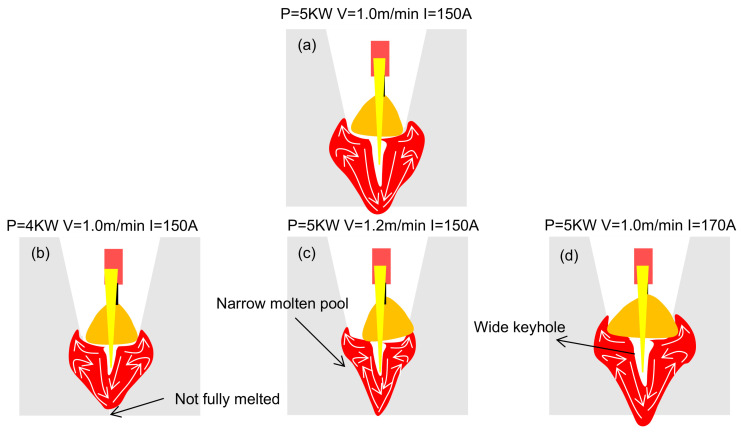
Flow behavior of the keyhole cross-section of the backing weld pool: (**a**) Cross-section of the molten pool at reference welding parameters; (**b**) Cross-section of the molten pool at low laser power; (**c**) Cross-section of the molten pool at faster welding speed; (**d**) Cross-section of the molten pool at higher welding current.

Figure 12 is a schematic diagram of the influence of different welding parameters on the flow behavior of the molten pool in the backing welding.

As shown in Figure 12a, the molten pool demonstrates an overall symmetric circular flow at the keyhole. The liquid metal flows along the keyhole to the bottom. It then flows up the slope wall and forms a cycle. The liquid metal near the surface fills both sides under the action of the Marangoni effect, and the weld width is limited by the U-shaped wall surface. Figure 12a,b show that when the laser power is insufficient to melt the base metal at the bottom, the flow range of the pool is small, which limits the expansion of the molten pool and the filling of the weld. Figure 12a,c show that when the welding speed is faster, the heat input per unit time is reduced, and the flow range size of the molten pool is significantly reduced. The accelerated cooling rate of the molten pool hinders the subsequent sufficient filling of the liquid metal, resulting in a worse filling effect on the side walls. Figure 12a,d show that increasing welding current helps to widen the keyhole and maintain the keyhole’s stability and enhances the flow within the molten pool. Welding current exceeding 170 A may cause the base metal to overheat, resulting in excessive melting of the bevel wall, which can adversely affect the quality of welds.

## 4. Conclusions

(1) The formation of porosity defects is primarily associated with the instability of the keyhole and the rapid solidification behavior of the molten pool. When the keyhole collapses under the action of surface tension, the gas inside is wrapped by the liquid metal, thus forming bubbles. While some bubbles escape upon the re-establishment of the keyhole, others remain trapped and migrate within the molten pool. Furthermore, the rapid solidification and high surface tension of the 4J36 Invar steel molten pool significantly impede bubble escape, ultimately forming porosity defects.
(2) The formation of porosity defects is influenced by the stability of the keyhole, the flow behavior of the molten pool, and the inherent properties of the material. Keyhole collapse is the cause of bubble generation. The flow of the molten pool is driven by recoil pressure, surface tension, and the Marangoni effect. Flow behavior influences the migration and escape of bubbles. Moreover, the fluidity of the molten pool of 4J36 Invar steel is poor, which further exacerbates the tendency of bubble retention and porosity formation. Due to its high tension and low fluidity, the 4J36 Invar steel molten pool exhibits a higher risk of porosity defects than 304 stainless steel.
(3) When the laser power is increased from 4 kW to 8 kW, the enhanced heat input significantly increases the penetration depth and enlarges the keyhole size. However, when the laser power exceeds 6 kW, it tends to induce keyhole instability. When the welding speed decreases from 3 m/min to 0.3 m/min, the prolonged thermal interaction promotes molten pool flow expansion. However, the welding speed exceeding 1.5 m/min increases porosity defects. Increasing the welding current from 100 A to 200 A widens the keyhole and enhances molten pool fluidity.
(4) The main measure to suppress porosity defects is the optimization of process parameters. To achieve a balance between molten pool fluidity and keyhole stability, the laser power, welding speed, and welding current are maintained at 4–6 kW, 0.5–1.0 m/min, and 150–170 A, respectively. The surface of the base material can also be cleaned to reduce the source of impurity gases, and the base material can be preheated to reduce the cooling rate of the molten pool.

## Figures and Tables

**Table 1:** Chemical composition of 4J36 Invar steel (wt.%).

Ni	C	Si	Mn	P	S	Fe
35.0–37.0	≤0.05	≤0.3	≤0.6	≤0.02	≤0.02	the rest

**Table 2:** Parameters of Invar steel welding experiment.

Welding Parameters of 10 mm Thick Invar Steel					
Serial Number	Laser Power (KW)	Welding Speed (m/min)	Welding Current (A)	Defocusing Amount (mm)	Shielding Gas Flow Rate (L/min)
1	6	1.2	100	0	20
Welding parameters of 20 mm thick Invar steel					
1	6	1	150	0	20
2	5	1	150	0	20
3	4	1	150	0	20
4	5	1.2	150	0	20
5	5	1	170	0	20

**Table 3:** Material properties of 4J36 and 304.

Material Properties	Unit	Numerical Size (4J36)	Numerical Size (304)
Solid-phase line temperature	K	1727	1727
Liquid-phase line temperature	K	1811	1723
Evaporation temperature	K	3200	3000
Surface tension	N·m^−1^	1.2	1.0
Density	kg·m^−3^	8130	6910
Latent heat of fusion	J·kg^−1^	2.74 × 10^5^	2.7 × 10^5^
Kinematic viscosity	kg·m^−1^·s^−1^	0.006	0.006
Solid-phase thermal conductivity	W·m^−1^·K^−1^	19	22
Liquid-phase thermal conductivity	W·m^−1^·K^−1^	70	34
Solid-phase specific heat capacity	J·kg^−1^·K^−1^	880	720
Specific heat capacity of the liquid phase	J·kg^−1^·K^−1^	920	800

**Table 4:** Different process parameters for molten pool simulation.

Serial Number	Laser Power (KW)	Welding Speed (m/min)	Welding Current (A)
1	4/6/8	3	150
2	4	0.3/1.5/3	100
3	6	1.2	100/150/200

## Data Availability

The original contributions presented in the study are included in the article, further inquiries can be directed to the corresponding author.
